# Modeling the transition state structure to probe a reaction mechanism on the oxidation of quinoline by quinoline 2-oxidoreductase

**DOI:** 10.1186/s13065-016-0219-3

**Published:** 2016-11-24

**Authors:** Enyew A. Bayle

**Affiliations:** Department of Chemistry, College of Natural and Computational Science, Haramaya University, Harar, Ethiopia

**Keywords:** Quinoline, Interaction site, Quinoline 2-oxidoreductase, Reaction mechanism

## Abstract

**Background:**

Quinoline 2-oxidoreductase (Qor) is a member of molybdenum hydroxylase which catalyzes the oxidation of quinoline (2, 3 benzopyridine) to 1-hydro-2-oxoquinoline. Qor has biological and medicinal significances. Qor is known to metabolize drugs produced from quinoline for the treatment of malaria, arthritis, and lupus for many years. However, the mechanistic action by which Qor oxidizes quinoline has not been investigated either experimentally or theoretically.

**Purpose of the study:**

The present study was intended to determine the interaction site of quinoline, predict the transition state structure, and probe a plausible mechanistic route for the oxidative hydroxylation of quinoline in the reductive half-reaction active site of Qor.

**Results:**

Density functional theory calculations have been carried out in order to understand the events taking place during the oxidative hydroxylation of quinoline in the reductive half-reaction active site of Qor. The most electropositivity and the lowest percentage contribution to the HOMO are shown at C_2_ of quinoline compared to the other carbon atoms. The transition state structure of quinoline bound to the active site has been confirmed by one imaginary negative frequency of −104.500/s and −1.2365899E+06 transition state energies. The Muliken atomic charges, the bond distances, and the bond order profiles were determined to characterize the transition state structure and the reaction mechanism.

**Conclusion:**

The results have shown that C_2_ is the preferred locus of interaction of quinoline to interact with the active site of Qor. The transition state structure of quinoline bound to the active site has been confirmed by one imaginary negative frequency. Moreover, the presence of partial negative charges on hydrogen at the transitions state suggested hydride transfer. Similarly, results obtained from total energy, iconicity and molecular orbital analyses supported a concerted reaction mechanism.

## Background

Quinoline 2-oxidoreductase is a member of molybdenum hydroxylases with a known three dimensional structure [1]. It catalyzes the oxidative hydroxylation of quinoline (2, 3 benzopyridine) to 1-hydro-2-oxoquinoline. Qor is known to oxidatively hydroxylate carbon atoms of heterocyclic aromatic compounds, particularly quinoline and its derivatives. For instance, it catalyzes the first two steps in the degradation of quinoline in bacteria (*Comamonas testosteroni* 63) [2]. Quinoline derivatives have been used in the treatments of malaria, arthritis, and lupus for many years [3]. They are also used as a sole source of energy in bacteria [1], hepatocarcinogen in mice and rats, and several quinoline derivatives are mutagens [4]. However, quinoline derivatives are known to represent one of the most successfully used classes of drugs, their therapeutic action is still not well understood. Remarkably, there is no clear catalytic mechanism known for the therapy of action of quinoline drugs [3]. Therefore, the catalytic mechanism of Qor needs to be investigated in order to improve the use of quinoline in the drug design process.

**Fig. 1:**
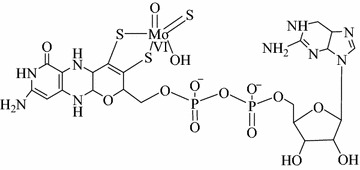
The chemical structure of the molybdenum cofactor (reductive half reaction) found in Qor Adopted from Ref. [1]

All molybdenum enzymes contain the molybdenum cofactor in common. The molybdenum cofactor is the reductive half-reaction active site of Qor [5]. It is composed of a Mo^(+VI)^ ion and a molybdopterin cytosine dinucleotide [5]. All ligands coordinated with molybdenum ion are inorganic ligands and the coordination adopts a distorted coordination sphere [1] (Fig. 1). It is labile in nature and highly sensitive to air oxidation as a result the chemical syntheses of either Moco or its intermediates have never been successful so far [5].

**Fig. 2:**
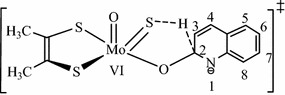
The general tetrahedral model structure used for predicting a transition state structure of the truncated Moco bound to quinoline, the numbers indicate the position of carbon atoms on quinoline

It was already known that molybdenum hydroxylases oxidatively hydroxylate their substrates at the electron deficient carbon center adjacent to nitrogen atom [6]. But, in the case of the oxidative hydroxylation reactions catalyzed by Qor, there are two ideas regarding to the interaction site of quinoline that interacts with the hydroxyl oxy-anion of the active site of Qor. Quinoline is proposed to have two interaction sites (Fig. 2). Some papers supported that quinoline interacts with its C_2_ with the active site [1, 2]. On the contrary, other investigations argued that quinoline interacts with the active site at its C_4_ position [6]. This discrepancy draws attention to probe the interaction site of quinoline. The overall reaction mechanism catalyzed by Qor is given in Eq. (1).

1 $$RH + H_{2} O \leftrightarrow ROH + 2H^{ + } + 2e^{ - }$$

where, R is the heterocyclic aromatic compounds [7]. Although some of the substrates and the corresponding products of the reaction catalyzed by Qor are known [6], the catalytic conversions of the reactants into the products and the events that are expected to takes place have never been described.

Qor catalyzes similar substrates with the enzyme Xanthine oxidoreductase (XOR) [6]. Quinoline, physiological substrates of Qor, and xanthine, physiological substrates of XOR, share some common features such as both are an aromatic compounds with two ring systems. Moreover, Qor and XOR are the members of molybdenum hydroxylases particularly xanthine oxidase family enzymes and hence basically they have similar redox active centers [7, 8]. For this reason the catalytic mechanisms of Qor is expected to be studied on the basis of the catalytic mechanisms of XOR [1]. XOR from bovine milk is the most studied members of molybdenum hydroxylase. Consequently, it can be used as a bench mark to study the entire members of Mo hydroxylase such as Qor [9]. Based on the currently accepted catalytic mechanisms of XOR [10], the catalytic mechanism of Qor is proposed in the study.

The reaction mechanism is proposed to begin with the abstraction of the equatorial hydroxyl proton by the amino acid residue (Glu743). The neucleophile, oxy-anion of the hydroxyl group, attacks the electron deficient carbon center of the substrate and provides a tetrahedral species (tetrahedral intermediate or transition state). At the transition state hydrogen is transferred from the substrate carbon to the sulfido terminal of the active site [11]. However, it not known whether oxidative hydroxylation of quinoline catalyzed by Qor is concerted or stepwise. In addition to that the mechanism of a catalytic reaction can be characterized in terms of the chemical events that take place during the reaction [12]. However, several events that are expected to occur during the oxidation of quinoline such as formation of a bond between the equatorial oxygen and the quinoline carbon, cleavage of quinoline carbon-hydrogen bond, migration of hydrogen from quinoline carbon to the sulfido terminal of the active site, and conversion of quinoline to 1-hydro-2-oxoquinoline were neither known nor described. Moreover the nature of hydrogen transfer from the substrate carbon to the sulfido terminal of Qor is not known.

**Scheme 1:**
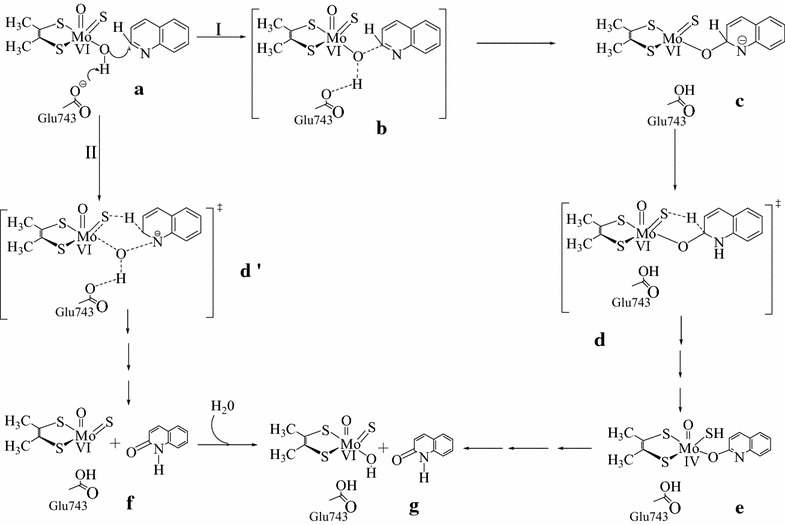
The hypothetical schematic model used to probe whether the catalytic oxidative hydroxylation of quinoline by Qor is stepwise (route, I) or concerted (route, II) Adapted from Ref. [10]

In order to probe either the concerted or stepwise mechanism, Scheme 1 is proposed for the oxidation of quinoline catalyzed by Qor. This hypothetical schematic model is expected to pass through the transition state structure (structured) for both the stepwise (route I) and concerted (route II) reaction mechanism. Moreover, at the transition state structure, hydrogen and electrons are expected to be transferred from the substrate carbon (C_RH_) to the sulfido terminal (S_Mo_) of the active site. However, the natures of proton and electrons transfer were not described.

A density functional theory approach was designed to perform electronic structure calculations in order to investigate the catalytic mechanism and describe the events those are expected to take place during the catalytic oxidative hydroxylation of quinoline by Qor. The calculations were performed on the truncated active site model compound bound to quinoline. From the optimized structures several data such as total energies, Mulliken atomic charges, bond distance, bond order indices, and percentage contributions of the chemical constituents to the molecular orbitals were generated. These data were used to determine the interaction site of quinoline, model the transition state structure, and probe a plausible mechanistic route for the oxidative hydroxylation of quinoline in the reductive half-reaction active site of Quinoline 2-oxidoreductase.

## Computational methods

The electronic structure calculations were performed with density functional theory method on the Gaussian® 03 W (version 6.0) program software package (Gaussian, Inc., Wallingford, CT, USA) [13]. The DFT method employing the B3LYP level of theory [14] was applied on the model structures derived from the initial geometries of the crystal structures of Qor [1]. The optimizations were carried out using the mixed basis set LANL2DZ for Mo which contains core potential (LanL2), and 6–31G (d^1^–p^1^) basis set for C, N, O and S [15].

The substrate quinoline and quinoline bound to the truncated reductive half-reaction active site of Qor at C_2_ and C_4_ position of quinoline were optimized in order to identify the interaction site of quinoline. The transition state structure was determined for the migration of substrate bound (H_RH_) from the substrate carbon (C_RH_) to the sulfido terminal (S_Mo_). The linear transit scans were performed on the structure shown on Fig. 2.

The transition state structure was located by the presence of one imaginary negative frequency [16]. The geometries from single point energy calculations were used for AOMix molecular analysis using AOMix 2011/2012 (reversion 6.6) software programs [17, 18]. The total energies and the Muliken atomic charges were generated from the optimized geometries of single point energy calculations. The total energies were normalized in order to profile the reaction coordinates.

**Scheme 2:**
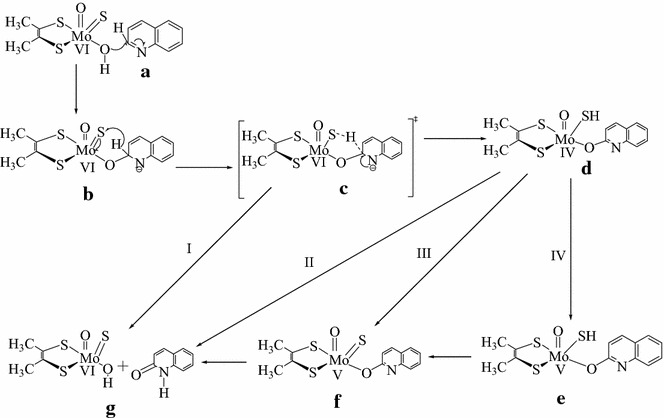
The geometries that were optimized to probe a reaction mechanism for the catalytic oxidative hydroxylation of quinoline by Qor (developed from scheme 1)

Moreover, the mechanistic routes for the oxidative hydroxylation of quinoline by Qor were probed by performing a series of geometry optimizations on the geometries shown in Scheme 2. The mechanistic routes were analyzed by describing the bonds that were formed and broken in terms of Muliken atomic charges, bond lengths, bond order indices, and the percentage contribution of the chemical constituents of to the molecular orbitals.

## Results and discussion

### Probing the interaction site of quinoline

**Fig. 3:**
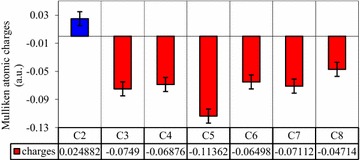
A plot of Muliken atomic charges, on the carbon atoms, obtained from the optimized structure of unbound quinoline. The position of the carbon atoms are indicated on Fig. 2

The Mulliken atomic charges on the carbon atoms of the unbound quinoline were calculated (Fig. 3). Accordingly, the data revealed the unique nature of one of the carbon atoms, C_2_, located in the pyridine ring. The C_2_-pyridine was shown to bear partial positive charges (0.025), the only atom with an electropositive charge. Unlike to this carbon atom, the remaining carbon atoms (in the benzopyridine ring) were shown to bear partial positive charges. According to the principle of nucleophilic/electrophilic reaction, nucleophiles prefer to attack the most electrons deficient species (carbon centers, in quinoline). Moreover, C_2_ and O_eq_ are oppositely charged which enables the equatorial oxygen to easily donate a pair of electrons to carbon (C_2_), an electrophile, to form a bond {[Mo^(+VI)^] O_eq_–C_2_-pyridine}.

**Fig. 4:**
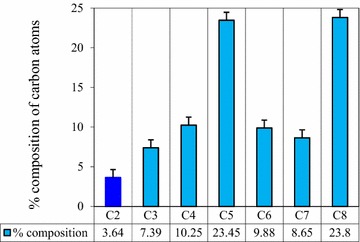
The percentage contribution of the carbon atoms to HOMOs of quinoline obtained from AOMix calculation

Similarly, the percentage contributions of the carbon atoms to the highest occupied molecular orbital (HOMO) of unbound (free) quinoline were calculated (Fig. 4). Accordingly, the lowest contribution to the HOMO is shown at C_2_-pyridine of quinoline. This reflects that the electron density on C_2_-pyridine is the lowest among the carbon atoms of unbound (free) quinoline. Even if the contribution on C_2_-pyridine is about 50% less than C_3_-pyridine, the preferred interaction site remains C_2_-pyridine.

Moreover, the total energies obtained from optimization for C_2_-quinoline or C_4_-quinoline bound to the active site (Mo^(+VI)^–O_eq_–C_2_–quinoline or Mo^(+VI)^–O_eq_–C_4_–quinoline, respectively) are (−1.23661074E+06) and (−1.23661438E+06)kcal/mol, respectively. These results clearly show that the active site bound at C_2_ position of quinoline is destabilized by 3.64 kcal/mol relative to the active site bound at C_4_ position of quinoline. This indicates that the active site bound at C_2_ position of quinoline exhibits lower energy barrier to enter the transition state compared to the active site bound at C_4_ position of quinoline.

Therefore, the data from Mulliken atomic charge profile, % contribution on HOMO, and total energies are in favor of C_2_-pyridine as the preferred interaction site for quinoline. The result is consistent with the previous findings that quinoline becomes hydroxylated at C_2_ atom of the heterocyclic nitrogen containing ring [1].

### Prediction and characterization of transition state structure

**Fig. 5:**
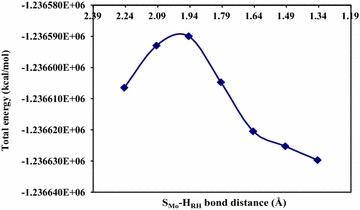
The total energy *plots* used to locate the initial guess geometry for the transition state structure search

The total energies from the linear transit scan calculation for quinoline bound to the reductive half-reaction active site of Qor are plotted as a function of S_Mo_–H_RH_ distance (Fig. 5). The total energy profile was used to locate the initial guess for the transition state structure. As a result, the initial guess for the transition state structure was assigned for the geometry with highest energy at S_Mo_-H_RH_ distances 1.946Å.

In addition to the total energies, the Mulliken atomic charges on selected elements (C_RH_, H_RH_, O_eq_, Mo, O_oxo_, S_Mo_, S_α_, and S_β_) from linear transit scan calculations were tabulated (Table 1).

**Table 1:** Mulliken atomic charges for selected elements from linear transit scan calculations

S_Mo_–H_RH_ (Å)	C_RH_	H_RH_	O_eq_	Mo	O_oxo_	S_Mo_	S_α_	S_β_
1.346	0.330	0.011	−0.469	0.414	−0.545	−0.391	−0.240	−0.221
1.496	0.323	−0.029	−0.467	0.428	−0.543	−0.367	−0.224	−0.219
1.646	0.328	−0.053	−0.469	0.428	−0.542	−0.359	−0.220	−0.215
1.796	0.335	−0.085	−0.473	0.428	−0.539	−0.353	−0.213	−0.207
1.946	0.199	0.048	−0.544	0.584	−0.532	−0.444	−0.184	−0.180
2.096	0.195	0.058	−0.562	0.618	−0.529	−0.474	−0.172	−0.172
2.246	0.084	0.142	−0.576	0.616	−0.567	−0.626	−0.220	−0.181

The Mulliken atomic charges on Mo are 0.616, 0.584, and 0.414 respectively, for the substrate bound intermediate, transition state, and product bound intermediate. This reflects a decrease in the partial positive charge on Mo ion as H_RH_ migrates from C_RH_ to S_Mo_. The decrease in charge on Mo indicates the development of negatively charged particles on it. This is consistent with the reduction of Mo as the substrate bound active site (Mo^(+VI)^) is converted to the product bound active site (Mo^(+IV)^). Unlike Mo ion, the Mulliken atomic charge on substrate carbon (C_RH_) was shown to increase as H_RH_ migrates from C_RH_ to S_Mo_. The charges on C_RH_ are 0.084, 0.198, and 0.330, respectively, at the substrate bound intermediate, transition state, and product bound intermediate. The profile reveals that the partial positive charges on C_RH_ was shown to increase by a factor of two as H_RH_ moves from the substrate bound carbon to the transition states and further increased by 66.3% as H_RH_ moves to the product bound intermediate. The increase in partial positive charge on C_RH_ is due to the partial transfer of electrons away from it. The increase and decrease in the partial negative charges on Mo and C_RH_ is consistent with the assumption that Mo is reduced from (Mo^(+VI)^) to (Mo^(+IV)^) in the course of the reaction, due to the transfer of electrons from C_RH_ the molybdenum center. Although the changes in magnitude are not comparable, the charge on the equatorial oxygen (O_eq_) shows the same trend as C_RH_. The atomic charge values on O_eq_ are −0.576, −0.544, and −0.469 when H_RH_ is at the substrate bound carbon, transition state, and product bound sulfido terminal, respectively. The decrease in the partial negatively charged particles on O_eq_ might be due to the increase in the attraction of bonding electrons (O_eq_–C_RH_) by C_RH_. On the other hand, the electropositivity of the substrate hydrogen (H_RH_) decreases as it moves from the substrate bound carbon to the product bound sulfido terminal. This indicates that the accumulation of negatively charged particles, on H_RH_, is high when it is found at the sulfido terminal compared to the substrate bound. Unlike all the other inorganic ligands coordinated to Mo, the atomic charge distribution on the apical oxygen shows no more significant variation as H_RH_ moves from C_RH_ to S_Mo_. As a result, it can be reasonably concluded that the apical oxo plays a “spectator” role in the reaction. In previous works, it was reported that the apical oxo may play an important role in the stabilization of the intermediate states of the catalytic cycle by increasing the Mo = O strength by “spectator oxo effect” though it is not directly participated in catalysis [1]. The charge distribution on H_RH_ at C_RH_–H_RH_, TS, and S_Mo_–H_RH_ are 0.142, 0.048, and 0.041, respectively. This result shows that the electropositivity of H_RH_ is decreased by 66.3% as H_RH_ move from C_RH_ to the transition state and further decreased by 76.1% at S_Mo_ compared to the transition state. The rapid decrease in electropositivity or rapid increase in electronegativity of H_RH_, as it migrates from C_RH_ to S_Mo_, is due to the development of partial positive charges on H_RH_. This result supported hydride transfer from C_RH_ to S_Mo_ which is consistent with recent investigations [20]. The partial negative charge distributions on the sulfido terminal (S_Mo_) are −0.626, −0.444, and −0.391 as H_RH_ is found at C_RH_, transition state, and S_Mo_ in the respective order. This result shows the increase in the electropositivity of S_Mo_ as H_RH_ moves from C_RH_ to S_Mo_ itself. This might be due to the transfer of partial negatively charged electrons from the π-type electrons between apical oxygen and molybdenum (Mo = O) to the empty d_xy_ orbitals of Mo. Finally, the atomic charge distributions on the dithiolene sulfurs slightly increase as H_RH_ moves from C_RH_ to S_Mo_. The result shows that the partial negatively charged particles are increased by 0.019 and 0.040 for S_α_ and S_β_, respectively. The increase in electronegativity might be due to the back donation of electrons from the d_xy_ orbital’s of Mo to the p_z_ orbitals of the dithiolene sulfur atoms. It implies that electrons from the Mo center passes to the other redox centers through the dithiolene sulfurs. The change in electronegativity of S_β_ is higher than S_α_ by 0.021. S_β_ is at about 150.134° angle from the equatorial oxygen which implies that S_β_ is almost trance to the equatorial oxygen. For this reason, S_β_, which carried the partial negatively charged particles, would have a trance effect on the equatorial oxygen which is a leaving group in the course of the reaction.

**Fig. 6:**
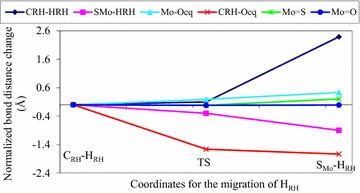
A plot for the normalized bond distance differences as a function of coordinates (C_RH_–H_RH_, TS, H_RH_) obtained from the linear transit calculation of quinoline bound with the reductive half reaction active site of Qor

Various bond lengths which are expected to be formed and broken while H_RH_ linearly moves from C_RH_ to S_Mo_ were collected from the out puts of the optimized structures. The optimized bond lengths versus the S_Mo_–H_RH_ bond distances were plotted (Fig. 6). The increase in the bond length of Mo–O_eq_ (Fig. 6) shows that the Mo–O_eq_ bond is broken as H_RH_ moves from C_RH_ to S_Mo_. On the contrary, the C_RH_–O_eq_ bond length is decreased as H_RH_ migrates from C_RH_ to S_Mo_. The shortening of C_RH_–O_eq_ bond length leads to the accumulation of electron density on the substrate carbon (C_RH_). The C_RH_–O_eq_ bond length is longer than Mo–O_eq_ bond length at C_RH_–H_RH_. However, the C_RH_–O_eq_ bond length is shorter than the Mo–O_eq_ bond length at the transition state. This result indicates that the C_RH_–O_eq_ bond is formed and the Mo–O_eq_ bond is broken before the transition state. The C_RH_–H_RH_ bond length is elongated unlike the S_Mo_–H_RH_ bond length which is decreased as H_RH_ moves from C_RH_ to S_Mo_. At the transition state, the C_RH_–H_RH_ bond length is lower than the S_Mo_–H_RH_ bond length. This shows that the transition state is more substrate like. Therefore, according to the Hammond’s principle, the transition state is early transition state. The Mo = S bond length is increased as H_RH_ moves from C_RH_ to S_Mo_. The increase in the Mo = S bond length shows the loss of the double bond character. This might be due to the delocalization of electrons between Mo and S_Mo_. Almost all the bond lengths of the atoms that are directly coordinated to the molybdenum metal center shows a significant change except Mo = O_oxo_ bond length which is almost constant whilst H_RH_ moves from C_RH_ to S_Mo_. This shows that the apical oxo plays a spectator role throughout the reaction and it is consistent with the results obtained from the atomic charges as described above.

In summary, results obtained from the bond lengths possibly predicts that the events which are proposed to takes place at the transition state such as bond formation (C_RH_–O_eq_ and S_Mo_–H_RH_) and bond cleavage (Mo–O_eq_ and C_RH_–H_RH_) inherit the characteristics of the substrate bound. Moreover, the lengthening of bond lengths predicts the cleavage of Mo–O_eq_ and C_RH_–H_RH_ bonds while the shortening of bond lengths predicts the formation of C_RH_–O_eq_ and C_RH_–H_RH_ bonds during the oxidative hydroxylation of quinoline in the reductive half-reaction active site of Qor.

The percentage contribution of the molecular orbital fragments (Mo_dxy_) to the highest occupied molecular orbitals (HOMOs) of Qor at the substrate bound C_RH_-H_RH_, transition state, and S_Mo_–H_RH_ are 2.17, 21.67 and 80.57, respectively. The result shows that the metallic character increase as H_RH_ moves from C_2_ of quinoline to S_Mo_. The increase in metallic character depicts that electrons are transferred from C_2_ of quinoline to the Mo center and hence the reduction of Mo^(+VI)^ to Mo^(+IV)^ during the oxidative hydroxylation of quinoline by Qor.

### Probing a reaction mechanism for the oxidation of quinoline

After the transition state structure was located, various geometries (Scheme 2) were optimized in order to understand the events which take place during the catalytic conversion of quinoline to 1-hydro-2-oxoquinoline and probe a plausible mechanistic route for the oxidative hydroxylation of quinoline in the reductive half-reaction active site of Qor. In this reaction mechanism the equatorial oxygen is proposed to nucleophilically attack the electron deficient carbon (C_2_) to form structure (b) after the deprotonation of the equatorial hydroxyl group of the active site by Glu713. The possible inorganic ligands that might be considered for the nucleophilic attack on C_2_ of quinoline are the equatorial oxo (O_eq_), apical oxo (O_oxo_) and sulfido terminal (S_Mo_).

The Mulliken atomic charge distributions on O_eq_, O_oxo_, and S_Mo_ of the active site before nucleophilic attack [at structure (a)] are −0.597, −0.468, and −0.415 (Table 2). This result shows that the accumulations of negatively charged particles on O_eq_ are higher than O_oxo_ and S_Mo_. It assures that O_eq_ is preferred for nucleophilic attack on C_2_ of quinoline. This is consistent with recent experimental results that the catalytically labile site should be O_eq_ coordinated with Mo rather than O_oxo_ [19]. On the other hand, the atomic charge on C_2_ of quinoline is 0.025 which shows that C_2_ and O_eq_ are oppositely charged as a result, electrostatic force of attraction would be experienced between O_eq_ and C_2_. Hence, O_eq_ can be nucleophilically attack C_2_ which is electron deficient and the reaction mechanism proceeds through nucleophilic attack on C_2_ of quinoline. In line with finding, X-ray structural analysis showed the lack of enough space for the substrate to approach the Mo center from the axial direction and hence O_eq_ is more reactive than O_oxo_ in the nucleophilic attack [20]. Hence, from this result it is reasonably concluded that O_eq_ is preferred for nucleophilic attacks on C_2_ of quinoline.

**Table 2:** The Mulliken atomic charges for selected elements from geometry optimization for the structures shown in Scheme 2

Geometries	C_RH_	H_RH_	O_eq_	Mo	O_oxo_	S_Mo_	S_α_	S_β_
a	0.020	0.117	−0.597	0.662	−0.468	−0.415	−0.153	−0.054
b	0.110	0.128	−0.586	0.633	−0.547	−0.644	−0.231	−0.182
c	0.200	0.049	−0.541	0.579	−0.520	−0.448	−0.187	−0.175
d	0.329	−0.006	−0.467	0.410	−0.524	−0.383	−0.238	−0.216
e	0.342	0.035	−0.526	0.528	−0.466	−0.235	−0.113	−0.079
f	0.349	–	−0.479	0.473	−0.522	−0.584	−0.205	−0.168

**Fig. 7:**
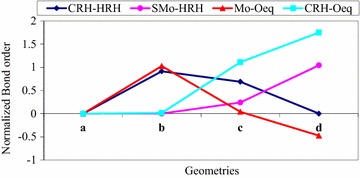
A *plot* of the normalized bond order for the active site structure bound to quinoline as a function of the respected geometries

After the nucleophilic attack, it is proposed that the Mo–O_eq_ and C_RH_–H_RH_ bonds are broken while C_RH_–O_eq_ and S_Mo_–H_RH_ are formed in the oxidative hydroxylation reaction mechanism as clearly described above. The formation and cleavage of these bonds are further proved by the results obtained from the bond order profiles (Fig. 7). The bond orders of Mo–O_eq_ and C_RH_–H_RH_ are decreased unlike C_RH_–O_eq_ and S_Mo_–H_RH_ as structure (b) is converted to structure (d) (Fig. 7). The decrease in the bonders of Mo–O_eq_ and C_RH_–H_RH_ assures the cleavage of these bonds in the reaction. On the other hand, the increase in the bond orders of C_RH_–O_eq_ and S_Mo_–H_RH_ predicts the formation of these bonds as structure (b) is converted to structure (d).

It is already described that the reaction mechanism can be proceed through nucleophilic attack by the equatorial oxygen on C_2_ and hydride transfer is taking place during the oxidative hydroxylation of quinoline. But, further description is requited whether the reaction mechanism is concerted or stepwise process.

**Fig. 8:**
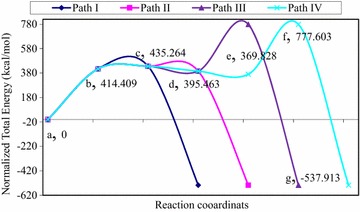
The total normalized energy of the four possible routes for the oxidation of quinoline in the active site of Qor

**Fig. 9:**
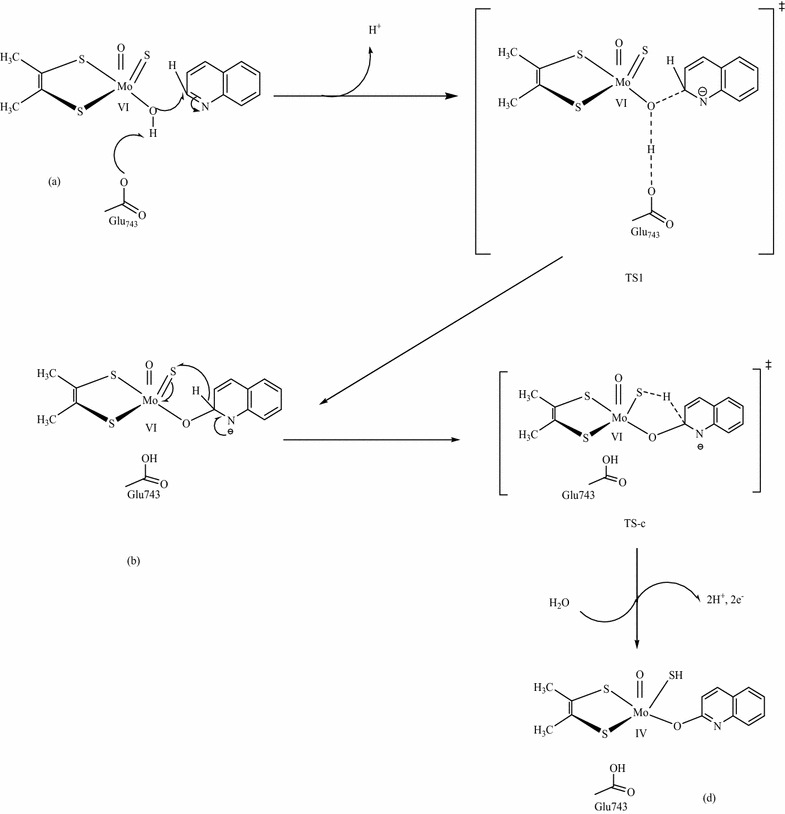
The proposed reaction mechanism to probe the feasibility of stepwise or concerted process for the oxidative hydroxylation reaction of quinoline in the active site of Qor

The normalized total energy differences between structure (b) (Mo^(+VI)^–O_eq_–C_RH_), which is formed as a result of nucleophilic attack on the substrate carbon, and the transition state [structure (c), (C_RH_…H_RH_…S_Mo_)^‡^] is 10.86 kcal/mol. This large energy difference indicates the difficulty of the conversion of structures (b) to (c) which argued the step wise process of nucleophilic attack. However, it is not sufficient evidence to conclude that the reaction mechanism is concerted. Therefore, it is better to compare the energy of structure (b) with the energy of the resting state geometry [structure (a)]. The normalized total energy difference between structure (a) and (b) is 414.41 kcal/mol (Fig. 8). This large energy barrier lets the existence of structure (b) under question unless there is a high energy species or intermediate between structures (a) and (b). Therefore, there might be a transition state structure (TS1) between structures (a) and (b) as shown in Fig. 9. For this reason, it is supposed that the abstraction of proton from the equatorial hydroxyl group of the active site by the amino acid residue (Glu743) and the nucleophilic attack of the equatorial oxygen on the substrate carbon are occurred simultaneously and coexist as transition state (TS1) between the resting state geometry [structure (a)] and the substrate bound intermediate [structure (b)]. In this case, the reaction would have two transition states designated as TS1 and TS-c (Fig. 9). The existence or inexistence of TS1 could be evaluated in comparison with TS-c.

**Fig. 10:**
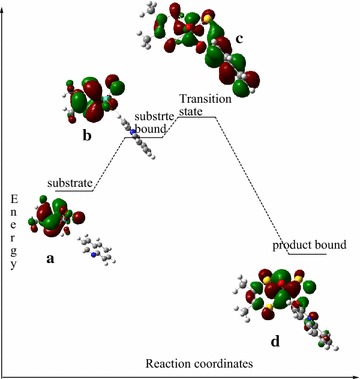
A *plot* of the energy of HOMOs as a function of the reaction coordinates for the oxidation of quinoline to 1-hydro-2-oxoquinoloine

The Mulliken atomic charges distribution on O_eq_ are −0.597, −0.586, and −0.467 at the structures (a), (b), and (d), respectively (Table 2). This indicates that the charge difference between structures (a) and (b) is 0.012 and for that of structures (b) and (d) is 0.119. The change in atomic charges on O_eq_ while structure (a) is converted to structure (b) is insignificant compared to the large charge difference observed when structure (b) is converted to structure (d). This large atomic charge differences between structures (b) and (d) is due to the presence of TS-c (hydride shift). Similarly, a comparable charge difference is expected if TS1 is found between structures (a) and (b). However, the result shows that there is no significant charge difference between structures (a) and (b). In addition to that, the atomic charge distribution on substrate carbon (C_2_) is also incomparable while structure (a) is converted to structure (b) and structure (b) is converted to structure (d). Once again, the charge difference between structures (a) and (b) (0.091) is insignificant compared to the charge difference between structures (b) and (d) (0.218). From this result, it can be concluded that the significance charge difference between structure (b) and (d) might be due to the presence of the transition state (TS-c). On the other hand, there is no significant charge difference between structures (a) and (b) which might be due to the inexistence of transition state (TS1). Therefore, transition state one (TS1) proposed for the reaction mechanism (Fig. 9) is not existed and the energy barrier between structures (b) and (c) is large (Fig. 10) which makes the conversion of structure (b) to structure (c) difficult. Hence, there is no intermediate [structure (b)] in the reaction mechanism.

In addition to that, there is no significant change in the percentage contribution of Mo_dxy_ to the HOMO as structure (a, 2.96) is converted to (b, 2.11). On the contrary, the conversion of structures (a) to (c, 20.96) or (c) to (d, 80.54) is takes placed with dramatic increase in the percentage contribution of Mo_dxy_ to the HOMO which assures the inexistence of structure (b) in the reaction mechanism. Similarly, the HOMOs in Fig. 10 show that there is no significant change in the electron densities distribution between structures (a) and (b). If structure (b) is existed in the reaction mechanism, there should be a change in the electrons densities distribution from structures (a) to (b) as the change shown from structures (a) to (c) and structures (c) to (d) in Fig. 10.

Once again, this result predicts that structure (b) is not existed in the reaction mechanism. Consequently the nucleophilic attack on the substrate carbon by the equatorial oxygen and the hydride transfer from the substrate carbon to the sulfido terminal of the active site are proposed to be concerted for the oxidative hydroxylation reaction mechanism of quinoline in the active site of Qor. This finding is consistent with theoretical and isotopic experimental results that a concerted (one step) mechanism by the deprotonated active site is the most plausible for reactions catalyzed by molybdenum hydroxylases [20].

Moreover, C_RH_–O_eq_ and C_RH_–H_RH_ bond lengths are changed from 1.452 to 3.137 and 1.201 to 1.091, respectively as H_RH_ migrates from the substrate bound [structure (b)] to the transition state (TS-c). This result indicates that the formation of C_RH_–O_eq_ bond is much higher (about 15 times) than the cleavage of C_RH_–H_RH_ bond. It implies that nucleophilic attack (C_RH_–O_eq_) is faster than hydride transfer (C_RH_–H_RH_). Hence, hydride transfer is the rate limiting step in the catalysis stage of the oxidative hydroxylation of quinoline in the reductive half-reaction active site of Qor. This result is consistent with previous findings that hydride transfer is the rate determining step in the concerted reaction mechanism unlike the stepwise mechanism in which the nucleophilic attack is the rate determining step [19].

After the product bound [structure (d)] is formed, it is further dissociated into various structures either through one or two electron transfer process to give the most stable product [structure (g)]. There are four possible paths (I, II, III and IV) for the dissociation of structure (d) into structure g (Fig. 9). Path (III) [(a), (c), (d), (f), and (g)] and path (IV) [(a), (c), (d), (e), (f), and (g)] are passed through the complex (f) which has 65.436 kcal/mol energy barrier from the transition state. Hence, path (III) and (IV) can be ruled out due to the highest energy barrier relative to path (I) and (II). Path (II) [(a), (c), (d) and (g)] has 39.801 kcal/mol energy barrier between the transition state [structure (c)] and the product bound [structure (d)]. On the other hand Path (I) [(a), (c), and (g)] is passed through the transition state and directly converted to the product (structure g). Due to this higher energy barrier (39.801 kcal/mol) relative to path (I), the reaction is not expected to pass through path (II). Therefore, the formation of the product [structure (g)] through path (II), (III), and (IV) will be retarded by 39.801, 65.436, and 65.436 kcal/mol respectively relative to path (I). In path (I), the product is formed with minimum energy relative to the other paths. Hence, path (I) is preferred for the product release stage for the oxidative hydroxylation of quinoline in the reductive half-reaction active site of Qor.

In summary, the results obtained from energy, charges, bond length, and percentage contribution of the chemical fragments to the HOMOs, and molecular orbital analysis supported concerted reaction mechanism for the oxidation of quinoline to 1-hydro-2-oxoquinoline on the in the reductive half-reaction active site of Qor.

## Conclusion

Density functional theory methods of electronic structures calculation was used for the study. Based on the data obtained from Mulliken atomic charge profile, % contribution on HOMO, and total energies, it is theoretically probed that C_2_ is the interaction site of quinoline.

The S_Mo_–H_RH_ bond distance for the model transition state structures of quinoline is found to be 1.960Å. The transition state structure was confirmed with one imaginary negative frequency of −104.5. The transition state total energy of quinoline is found to be −1.2365899E+06 kcal/mol.

The increase and the decrease in the partial positive charges on Mo and C_2_ of quinoline shows that molybdenum is reduced from Mo^(+VI)^ to Mo^(+IV)^ in the course of the reaction due to the transfer of electrons from C_2_ of quinoline to the molybdenum center. Likewise, the partial negative charge on O_eq_ is decreased due to the withdrawal of bonding electrons (O_eq_–C_RH_) away from it. On the other hand, the electropositivity of the substrate hydrogen (H_RH_) is decreased due to the accumulation of negatively charged particles on it. The apical oxo plays a “spectator” role in the reaction as it shows insignificant charge variations. Moreover, the equatorial oxygen is a better nucleophile relative to the apical oxo since the accumulation of partial negative charge on the equatorial oxygen is higher than the apical oxo.

The increase and the decrease in the bond lengths predicted the cleavage of Mo–O_eq_ and C_RH_-H_RH_ and formation of C_RH_–O_eq_ and S_Mo_–H_RH_ bonds at the transition state, respectively. The increase in metallic character of molybdenum revealed that electrons are transferred from C_2_ of quinoline to Mo center and hence the reduction of Mo^(+VI)^ to Mo^(+IV)^ during the oxidative hydroxylation of quinoline by Qor.

From the Mulliken atomic charge changes, it is reasonably predicted that the equatorial oxygen is a better nucleophile in the oxidative hydroxylation of quinoline. The decrease and the increase in the partially negatively charged particles on Mo and C_2_, respectively assured the transfer of electrons from C_2_ of quinoline to the Mo center. The accumulation of partial negative charges on the hydrogen atom at the product bound relative to the substrate bound, possibly predicted that hydrogen is transferred in the form of hydride (H + 2e^−^) from C_2_ to S_Mo_. Eventually, it is reasonably concluded that the oxidative hydroxylation of quinoline in the reductive half-reaction active site of Qor are concerted.

## Figures and Tables

**Fig. 11:**
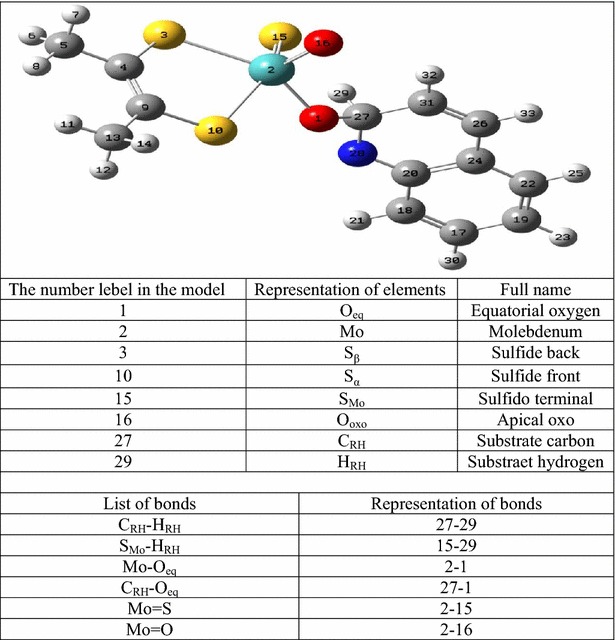
The descriptions for the abbreviations: C_RH_, H_RH_, O_eq_, Mo, O_oxo_, S_Mo_, S_α_, S_β_, C_RH_–H_RH_, C_RH_–O_eq_, S_Mo_–H_RH_, Mo–O_eq_, S_Mo_–H_RH_, Mo–S, and M=O

## Appendix

See Fig. 11.
